# Subject-Specific Alignment and Mass Distribution in Musculoskeletal Models of the Lumbar Spine

**DOI:** 10.3389/fbioe.2021.721042

**Published:** 2021-08-31

**Authors:** Marie-Rosa Fasser, Moritz Jokeit, Mirjam Kalthoff, David A. Gomez Romero, Tudor Trache, Jess G. Snedeker, Mazda Farshad, Jonas Widmer

**Affiliations:** ^1^ Department of Orthopaedics, Balgrist University Hospital, Zurich, Switzerland; ^2^ Institute for Biomechanics, ETH Zurich, Zurich, Switzerland

**Keywords:** spine biomechanics, musculoskeletal modelling, subject-specificity, upper body mass distribution, thoracolumbar alignment, automatized model generation, spine loading prediction, bi-planar radiography

## Abstract

Musculoskeletal modeling is a well-established method in spine biomechanics and generally employed for investigations concerning both the healthy and the pathological spine. It commonly involves inverse kinematics and optimization of muscle activity and provides detailed insight into joint loading. The aim of the present work was to develop and validate a procedure for the automatized generation of semi-subject-specific multi-rigid body models with an articulated lumbar spine. Individualization of the models was achieved with a novel approach incorporating information from annotated EOS images. The size and alignment of bony structures, as well as specific body weight distribution along the spine segments, were accurately reproduced in the 3D models. To ensure the pipeline’s robustness, models based on 145 EOS images of subjects with various weight distributions and spinopelvic parameters were generated. For validation, we performed kinematics-dependent and segment-dependent comparisons of the average joint loads obtained for our cohort with the outcome of various published *in vivo* and *in situ* studies. Overall, our results agreed well with literature data. The here described method is a promising tool for studying a variety of clinical questions, ranging from the evaluation of the effects of alignment variation on joint loading to the assessment of possible pathomechanisms involved in adjacent segment disease.

## 1 Introduction

The high incidence of back pain in the general population poses a socio-economic burden on society (Traeger et al., 2019; Hoy et al., 2014; Dagenais et al., 2008). Despite the increasing number of treatment options, self-assessed patient satisfaction stagnates (Friedly et al., 2010). This motivates the investigation of spinal biomechanics with the intention to improve diagnosis, treatment, and rehabilitation options (Widmer et al., 2020). Developing preventive measures and suitable treatment strategies for spinal pathology implies knowledge about the loading conditions within the spine and its muscles. The effect of physiologically pertinent mechanical loading conditions on various spinal tissues has been thoroughly studied *in vivo* (Polga et al., 2004; Daggfeldt and Thorstensson, 2003; Wilke et al., 2001; Sato et al., 1999) and *in vitro* (Rohlmann et al., 2009, 2001; Wilke et al., 2003). Although providing valuable insight into the spinal loading response, several disadvantages come along with experimental studies. The invasiveness of *in vivo* measurements raises ethical concerns with respect to both healthy and pathological subjects. *In vitro* experiments allow the investigation of loading patterns in a well-controlled environment, but the lack of muscular activity acts as a limiting factor. To overcome constrained sample availability and variability, musculoskeletal models have been established as a non-invasive alternative to study the intricate processes in the healthy and pathological spine (Christophy et al., 2012; Bruno et al., 2015; Senteler et al., 2017). These multi-rigid body models can be used to simulate the neuromuscular activity of the human body through inverse kinematics and static optimization, providing muscle forces and joint loads as an output. Musculoskeletal models can be used to investigate the loading conditions and optimal posture during physiological activities, e.g., in the context of preventive or rehabilitative exercises. Furthermore, the use of these models has the potential to improve pre-operative planning by not only taking geometrical aspects into account but also by considering functional aspects. In addition to valuable direct information about spine loading, the output of a robust model can enhance patient-specificity in other modeling modalities, e.g., providing more physiological loading conditions in finite element models (Esat and Acar, 2004; Toumanidou and Noailly, 2015).

Thus far, a variety of musculoskeletal models with increasing complexity has been introduced in the literature (Damsgaard et al., 2006; de Zee et al., 2007; Delp et al., 2007; Christophy et al., 2012; Bruno et al., 2015; Malakoutian et al., 2018; Ignasiak et al., 2016a). Existing models that are validated against *in vivo* measurements, like the implementations by Christophy et al. (2012) and Bruno et al. (2015) serve as important references for the development of new approaches. However, these models are generic, based on data from few individuals, or they are a statistical representation of specific cohorts of people. Although it was shown that properties such as spinopelvic alignment, weight, and height affect the loading at intervertebral joints (Senteler et al., 2014; Han, 2013; Caprara et al., 2020), the extensive variability amongst individuals within the human population is hardly captured. This necessitates new modeling approaches that include individualized spinopelvic alignment and mass distribution. Furthermore, patient-specific model creation is tedious and time-consuming. For successful incorporation into the clinical workflow, subject-specificity, as well as automation of the process, is called for. Bassani et al. (2017) presented the first attempt towards semi-automatic model creation from annotated bi-planar x-ray images. However, the positioning of the center of mass for each segment was based on earlier literature findings. Building on this idea and previous research, the present work focuses on patient-specific scaling and alignment of the spinal geometry as well as an individualized mass distribution. To control all steps from geometric morphing to minimization of the quadratic muscle activity, the model was implemented in MATLAB, a programming framework widely adopted in the research community.

Overall, the aim of this work was to develop and validate a pipeline for the creation of semi-subject-specific musculoskeletal simulations which provides great flexibility in terms of future research questions to be studied. The following sections give a detailed description of the model’s features and present results as well as the validation thereof. Subsequently, the advantages and limitations of the presented modeling approach are discussed.

## 2 Materials and Methods

All steps associated with model generation, simulation, and results analysis were automatized and carried out with custom-written scripts in MATLAB (R2020b, TheMathWorks Inc., Natick, MA, United States).

### 2.1 Model Generation

#### 2.1.1 Image Annotation

**FIGURE 1:**
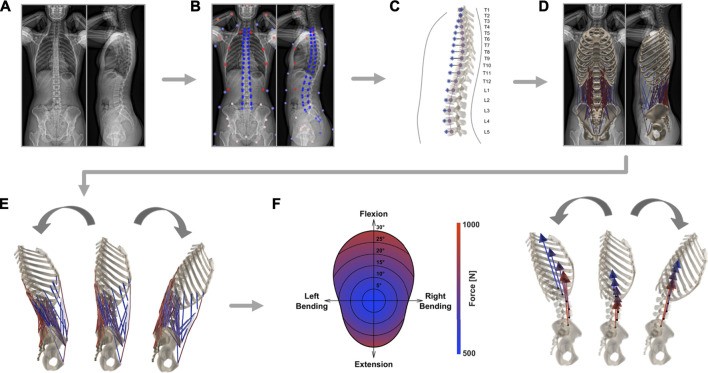
Schematic depiction of the steps required for the generation of individualized musculoskeletal models. After EOS image acquisition **(A)**, the bi-planar radiographs were annotated by an experienced medical professional **(B)**. From the resulting landmarks, the alignment of the thoracolumbar spine (purple) and the segment-wise position of the center of mass (blue) were derived **(C)**. A model rendering subject anatomy was created and included the eight major muscle groups involved in stabilizing the lower spine **(D)**. Kinematic boundary conditions were set **(E)** and consequently magnitude and direction of joint load was computed based on static optimization **(F)**.

First, a defined set of anatomical landmarks are identified on bi-planar radiography images (EOS imaging, Paris, France; Figure 1A). In total, 112 and 109 points are marked on the frontal and sagittal planes, respectively. Annotated structures are the thoracic and lumbar vertebrae, the sacrum, the pelvis, the femoral head, the rib cage, and the body outline, as well as head and arms (Figure 1B). Thanks to the spatial calibration of the EOS system, the 2D anatomical landmarks derived from the simultaneously acquired orthogonal images can then be converted into 3D coordinates.

#### 2.1.2 3D Model and Alignment

The proposed musculoskeletal model consists of seven functional segments: the rib cage, the five lumbar vertebrae, and the sacropelvic bone structures. Generic template models of vertebrae, ribs, sternum, pelvis, and sacrum are scaled and repositioned according to size and alignment represented in EOS images.

First, the coordinates of the vertebral body endplates (i.e., the four corners of the vertebral body detectable in the sagittal plane and four corners discernable in the frontal plane) are used to determine the scaling factors along all three axes of each vertebra. The width of the vertebra is scaled based on information from the frontal view image and height and depth, i.e. size in anteroposterior direction, are obtained from sagittal images.

The same landmarks are then used to fit a cubic spline through the centers of the vertebrae, from the uppermost thoracic level all the way down to the coccyx (Figure 1C). The resulting best-fit curve describes the patient-specific alignment of the spine and allows to keep the relative position of each bony segment constant (independently from the specifications resulting from imposed kinematics, Section 2.2). Correct arrangement of the scaled vertebral surface models along the spline is ensured by positioning the centroids of the template vertebral bodies on the respective centroids on the spline. Additionally, the vertebrae are rotated to align them with the orientation derived from landmarks in the sagittal and frontal planes. Next, the sternum and ribs are scaled and repositioned according to the location of the vertebrae and annotations of the ribcage (left, right, and anterior outline). Furthermore, pelvis and sacrum sizes are scaled to subject-specific dimensions based on the annotations of the femoral heads, the center of the sacral endplate, and the anterior superior iliac spines. The landmarks associated with the latter structures are used to rotate the pelvis and the sacrum in the frontal plane. The alignment within the sagittal plane relative to the longitudinal body axis is determined based on the vector from the center of the sacral endplate to the femoral heads for the pelvis and the vector to the caudal end of the coccyx for the sacrum. To achieve even better correspondence between 3D model geometry and the actual subject anatomy, the template pelvic bone is finally morphed onto the subject-specific landmarks using the *As-Rigid-As-Possible*-algorithm by Sorkine and Alexa (2007) (Figure 1D).

The alignment and dimensions of the thoracolumbar vertebrae within the sagittal and coronal plane (lumbar lordosis, thoracic kyphosis, sagittal vertical axis) are replicated in order to have a consistent placement of the center of masses, muscle attachment points, and center of rotations, which are all highly dependent on the subject’s anatomy. The scaling and positioning of the sacropelvic components accurately reproduce the subject’s anatomy and alignment (sacral slope, pelvic tilt, pelvic incidence) according to landmarks of the sacral endplate, the femoral heads, and the pelvis.

#### 2.1.3 Mass Distribution

We use the body contour obtained from the bi-planar EOS scans to determine the position of the center of mass (COM) for each relevant segment. The sagittal image is used to determine the body delimitation towards the anterior and posterior and the coronal image is used to determine the left and right outline of the torso. Seven regularly-spaced landmarks are positioned along each of the outlines (anterior, posterior, left, and right, Figure 1B). Through each set of landmarks, a spline function is fitted to obtain a smooth and continuous body demarcation (Figure 1C). The torso is subdivided into seventeen segments, each associated with one thoracolumbar vertebra. Every segment is then further subdivided into 2 mm thick elliptically-shaped slices. For each body segment, the corresponding center of volume (COV) is computed as a mean of the COVs of all 2-mm slices contained in the segment. Homogeneous density distribution at each level was assumed. Therefore, the COM of the segments coincides with the COV in our models. The mass assigned to each level is based on experimentally derived percentage distribution (Pearsall et al., 1996). The COM of the ribcage is lumped to a single point computed from the COMs of the twelve thoracic segments, the head, and the arms, weighted by the respective percentage mass contribution (Pearsall et al., 1996; Bruno et al., 2015). These weighting parameters, together with the estimation of the volume and the experimentally derived mean values for density at each level, are used to extrapolate the body weight (BW) of the subjects (Pearsall et al., 1996). We tested the procedure for BW estimation with a dataset comprising 82 subjects with available bi-planar radiographs and of known weight (mean weight being 77 kg, ranging from 43 to 135 kg; unpublished data). The correlation between measured and predicted body weight was high (Pearson’s correlation: *ρ* = 0.89, *p*-value > 0.0001). The mean absolute prediction error (MAE) was 7.0 Kg and the mean absolute percentage error (MAPE) was 9.0%.

#### 2.1.4 Joints and Muscles

The single rigid parts of the musculoskeletal model are connected through spherical joints, with the sacropelvic bone being fixed in space. The resulting six centers of rotation (COR) connecting the segments to each other are positioned in the middle of the respective intervertebral space. Each of the 230 model’s muscle fibers is assigned to one of the following eight muscle groups: external abdominal oblique, internal abdominal oblique, latissimus dorsi, psoas major, quadratus lumborum, rectus abdominis, erector spinae, or multifidus. Every muscle fiber connects two or more rigid components. Muscle attachment points and muscle properties (pennation angle, optimal fiber length, tendon slack length, maximal isometric force) are implemented based on previously published generic models (Christophy et al. (2012); Bruno et al. (2015), Figure 1D). Consistent placement of insertion points is possible by defining them relative to the nodes of the template meshes. The displacement-dependent behavior of muscle fibers is described with a simplified Hill-model (Hill, 1938), where only the active force contribution of the fibers is modeled. A Gaussian function was used to describe the active force-length relationship (Thelen, 2003). The optimal fiber length of each muscle fiber was taken from Bruno et al. (2015) and scaled according to the ratio between the original resting length and the subject-specific resting length. The latter was computed after each muscle attachment point being positioned according to the scaled, translated, rotated, and morphed rigid body.

### 2.2 Model Analysis

Inverse kinematics allows to derive joint reaction forces (JRF) and muscle activation patterns based on prescribed displacements and imposed external loads. The segmental motion constraints for the rigid bodies are obtained from *in vivo* measurements (Widmer et al. (2019), Table 1). The tabularized values indicate the percentage contribution of each segment to prescribed overall rotation in the sagittal (flexion and extension), frontal (lateral bending), and transverse (axial rotation) plane (Figure 1E). The overall angle of rotation was measured between the thorax and the fixed sacrum.

**TABLE 1:** Mean values of segment contribution to overall lumbar range of motion in flexion, extension, lateral bending, and axial rotation. The mean values of several *in-vivo* measurements are shown and were obtained from Widmer et al. (2019).

Segment	Flexion (%)	Extension (%)	Lateral bending (%)	Axial rotation (%)
L1L2	14	27	22	20
L2L3	19	21	26	23
L3L4	21	12	25	20
L4L5	25	9	17	20
L5S1	21	31	10	17

To compute the JRFs and muscle activity, a static optimization approach is employed (Figure 1F). This necessitates the construction of the moment equations for each joint comprising active contributions from muscles, forces derived from body mass distribution, and the reaction force from the more cranially positioned joints. Due to the high number of actuators (muscle fibers) with respect to the degrees of freedom, an infinite number of solutions available to reach equilibrium exists. To reduce the space of possible solutions, maintenance of energy efficiency in human muscle activation is assumed (Hicks et al., 2015). This allows to solve the moment equilibrium by minimizing the squared sum of muscle activity, which was set to range between 0.01 and 1:

$$C=\overset{m}{\underset{i=1}{\sum }}{a_{i}}^{2},\;0.01\leq a_{i}\leq 1$$ (1)

where *C* is the cost function, *a*_*i*_ is the activation of the muscle fiber *i*, and *m* is the total number of muscle fibers. Minimization of this cost function was achieved with the *Interior point* optimization algorithm embedded in the *fmincon* MATLAB function and the initial guess for muscle activity *a*_0,*i*_ was set to 0.5 for all fibers. A muscle fiber activity of 0.01 is implemented as lower boundary for the optimization to partially compensate for neglecting muscle co-activation with the use of the cost function from Eq. (1). The neutral posture (0° position) was set to the point of minimum load of pre-run simulations of pure flexion-extension movements.

### 2.3 Dataset

To test the procedure presented in the previous sections, a dataset comprising bi-planar radiography images of 145 subjects (76 females, 69 males) was examined. The images were acquired at Balgrist University Hospital between June 2012 and November 2020. Exclusion criteria were the presence of implants in the vicinity of the spine and scoliosis in the thoracolumbar region [Cobb’s angle ≥10°, Cobb (1948)]. The anonymized images were annotated by a medical professional using a custom graphical user interface. Based on the landmarks from the annotated images, the lateral spinopelvic parameters were computed for all subjects. Determination of pelvic incidence, sacral slope, and pelvic tilt followed the description expounded in Legaye et al. (1998). The sagittal vertical axis was defined as the horizontal distance between the plumb line and the posterior corner of the sacral endplate. The thoracic kyphosis angle was measured between the superior endplate of T1 and the inferior endplate of T12. Correspondingly, lumbar lordosis described the angle between the superior L1 endplate and the sacral endplate. An overview of the demographic data and the postural measurements is presented in Table 2.

**TABLE 2:** Mean, standard deviation, and range (minimum-maximum) are specified for age, weight, and spinopelvic parameters of the subjects included in this study. Except for age, all the information were computed based on annotated EOS images.

	Mean	Standard deviation	Range (min−max)
Age (Years)	39	24	7–85
Weight (kg)	66.5	22.7	18.8–137.0
Pelvic Incidence (°)	46.9	12.9	15.4–89.2
Sagittal Vertical Axis (mm)	6.6	35.6	−96.4–124.6
Sacral Slope (°)	34.4	10.2	10.4–64.1
Pelvic Tilt (°)	12.5	9.0	−11.1–37.6
Lumbar Lordosis (°)	50.3	13.7	0.8–83.2
Thoracic Kyphosis (°)	33.1	11.4	5.5–63.6

Next, individualized musculoskeletal models were created for each subject following the procedure described in Section 2.1. Consequently, the muscle activity and the intersegmental load were evaluated through static optimization in standing position and during flexion (maximal 30°), extension (maximal 20°), lateral bending (maximal 20°), axial rotation (maximal 30°), as well as for combinations of angles in the transversal plane (Section 2.2).

The consistency of the landmark positioning (Section 2.1.1) was assessed by quantifying the intra-rater and inter-rater reliability of annotations with intraclass correlation coefficients (ICC). Alignment parameters, weight estimation, and representative model results, such as the magnitude of the joint loads integrated over all levels and the summed tension generated by all lumbar erector spinae muscle fibers (in neutral position), were compared. One rater annotated a set of images at two different time points (TT), while another rater annotated the same set of images once (MRF). Annotations from nineteen images were considered for ICC of the alignment parameters and weight estimation, while the annotations from five different images were used to compare the reliability of the obtained model results. The radiographs for reliability evaluation were randomly selected from the available 145 images.

The compression and the anteroposterior shear components of the joint load are computed based on the local coordinate system linked to every joint. The compression component acts along the local axial direction, which is defined by the vector linking the considered joint and the joint next to it in cranial direction. The anteroposterior shear acts along the axis within the sagittal plane that is perpendicular to the local axial direction. A positive anteroposterior shear component indicates a contribution towards the posterior vertebral structures.

### 2.4 Validation

To validate the overall modeling approach, the results computed for our subjects were compared with those obtained by various *in vivo* and *in situ* studies (Lund et al., 2012; Hicks et al., 2015; Galbusera and Wilke, 2018). Information about the published studies used for validation are summarized in Supplementary Table S1. Several upper body postures were simulated for the comparisons mentioned below: standing in a neutral position, flexion (30°), extension (15°), lateral bending (20°), and axial rotation (30°). For lateral bending and axial rotation, the average between the movement to the left and to the right was considered.

First, measurements in patients who had a telemeterized vertebral body replacement implanted at the L1 level (Rohlmann et al., 2008) were compared to the results of previously published musculoskeletal models (Han et al., 2012; Bruno et al., 2015) and to the outcome of our analysis. For this purpose, the average compressive joint reaction forces at the L1L2 joint of the entire cohort were considered, as well as the simulated load in a single subject (male, 74 years, 69 Kg) with weight and age properties matched to the experimental conditions (2 males, 62 and 71 years, 66 and 72 Kg). Results for flexion, extension, lateral bending, and axial rotation were normalized to upright standing.

Next, the intradiscal pressure (IDP) measured within the L4L5 disc of healthy subjects in three different *in vivo* studies (Wilke et al., 2001; Sato et al., 1999; Takahashi et al., 2006) was compared to the outcome of the simulations. To account for a varying (mean) cross-sectional area (CSA) of the L4L5 IVD in the different experimental studies, the various experimentally determined IDPs were multiplied by the respective CSA. This output could then be compared to the compression force acting on the L4L5 joint in the musculoskeletal models after adjusting for the relationship between IDP and compressive JRF (*F*_*c*_) with the following published equation:

$$IDP\cdot CSA_{IVD}=\frac{F_{c}}{f},\;\left[N\right]$$ (2)

where *CSA*_*IVD*_ is the CSA of the IVD and the factor *f* was set to 0.66 according to literature findings (Nachemson, 1959; Bruno et al., 2015). The compared upper body positions between measurements and simulation results depended on the available experimental data (Wilke et al. (2001): standing, flexion, extension, lateral bending, and axial rotation; Sato et al. (1999): standing, flexion, extension; Takahashi et al. (2006); standing, flexion). Both, the average cohort results, as well as the results for a subject (male, 34 years, 74 Kg) with characteristics comparable to the experiments (Supplementary Table S1), were analyzed.

Finally, a segment-wise comparison of the magnitude of compressive forces during standing was performed between other modeling studies (Ignasiak et al., 2016a; Bassani et al., 2017; Bruno et al., 2017) and the current one. In the work of Bruno et al. (2017) musculoskeletal models were generated for 125 male subjects with broad ranges of alignment, weight, and age parameters (Supplementary Table S1). We compared the results of their models (scaled by subject weight and height, and incorporating subject-specific rendering of the spine curvature) with the predicted load acting on the joints of the subject-specific models generated for the current study.

## 3 Results

### 3.1 Spinal Alignment and Mass Distribution

**FIGURE 2:**
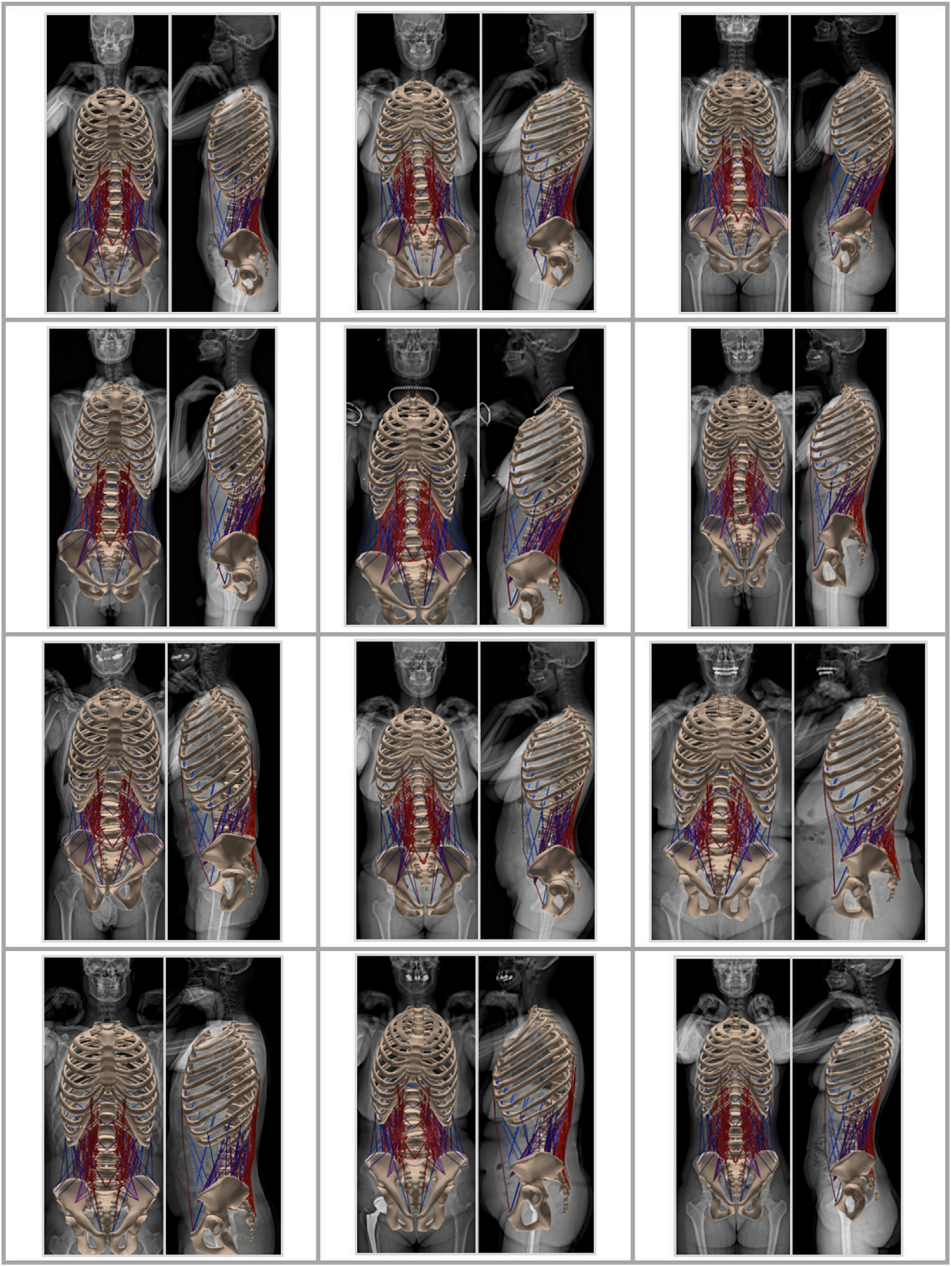
Musculoskeletal models generated based on the EOS images of 12 subjects.

**FIGURE 3:**
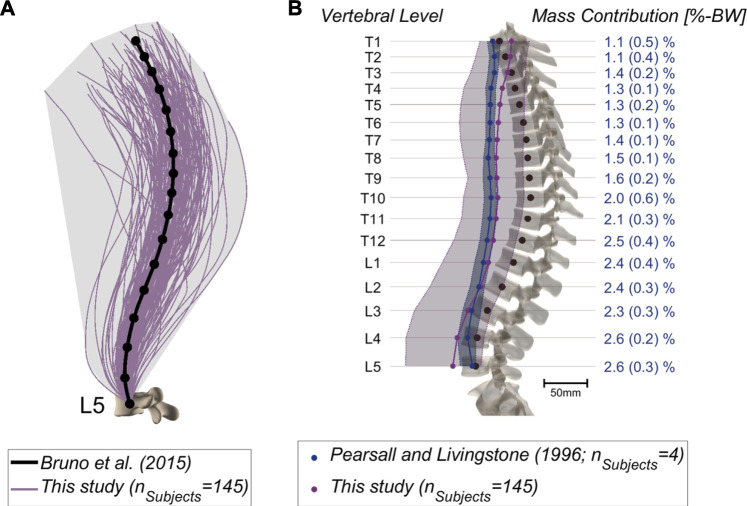
**(A)** Alignments of the thoracolumbar spine derived from 145 patients based on cubic splines fitted through the vertebral centroids. Emphasis lies on the large variation compared to a generic model [Bruno et al. (2015); anatomy based on 25-years-old male, 50th percentile for height and weight, thoracic and lumbar curvature angles from average measurements]. **(B)** COM position relative to the vertebral centroids in the sagittal plane. The mean and range of the values (minimum-maximum) are depicted for the CT-derived measurements (blue) (Pearsall et al., 1996) and for our dataset (purple). Further, the percentage body weight concentrated at each segment was derived from literature and is indicated on the right (Pearsall et al., 1996).

Musculoskeletal models were successfully generated based on the bi-planar images of 145 subjects (Figure 2). The intra-rater ICCs were computed to quantify the reliability of landmark positioning by a single rater at different time points in terms of the consistency of the obtained results (alignment, weight, and simulation results). All ICCs were greater than 0.90, except for those associated with the lumbar lordosis (ICC: 0.89; 95%: CI 0.74-0.96), pelvic incidence (ICC: 0.83; 95%: CI 0.60-0.93), and the sacral slope (ICC: 0.72; 95%: CI 0.41-0.88). Similar values were obtained for the assessment of inter-rater reliability, i.e., the comparison of annotations performed by two different raters (all ICC values and associated 95% CI are reported in Supplementary Table S2). Figure 3A depicts the thoracolumbar alignment of all subjects in the sagittal plane and with respect to the centroid of the L5 vertebra. As suggested by the values listed in Table 2, there are substantial variations amongst the curves and regarding the single alignment studied by Bruno et al. (2015). The average distance of the COM from the centroid of each vertebra diverged from previously reported computed tomography (CT)-derived measurements, particularly in the upper thoracic region and the lower lumbar spine (Pearsall et al., 1996, Figure 3B). The maximum relative distance towards the anterior from the vertebral center to the COM was determined at the L3 level with a magnitude of 85 mm, while a distance of 35 mm towards the posterior was measured at the uppermost thoracic vertebra.

### 3.2 Joint Reaction Forces

**FIGURE 4:**
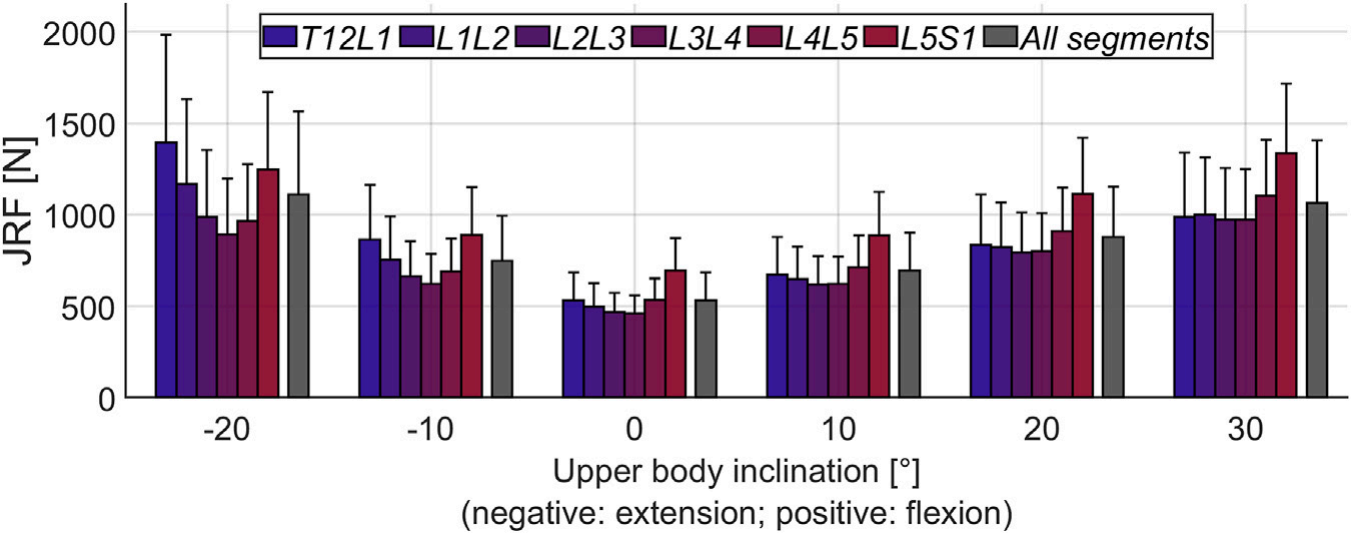
Mean magnitude of JRFs during flexion-extension movement of the upper body. *All segments* refers to the average loading across the six considered joints (T12L1 to L5S1). The error bars indicate the standard deviation.

**FIGURE 5:**
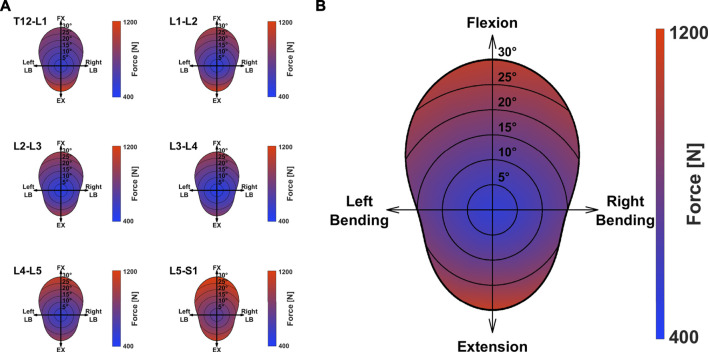
Mean magnitude of JRF during movement around the axes perpendicular to the caudo-cranial axis. Values for the single segments **(A)** and the average loading over all considered joints **(B)** are depicted. FX: Flexion; EX: Extension; LB: Lateral Bending.

Figure 4 depicts the mean JRFs at different levels for the studied cohort (magnitude of compression and shear components are shown in Supplementary Figure S1). During flexion, the maximum load was found at the L5S1 joint, whereas extreme extension led to the highest joint loads at the T12L1 level. The heatmap-representation in Figure 5 shows static optimization results as a mean for all subjects (segment-wise in Figure 5A and average of all levels in Figure 5B). It encompasses angular rotations around the axis normal to the sagittal plane (30° to −20°) and rotations along the axis normal to the frontal plane (15° to −15°), as well as combinations thereof. In general, the highest forces were observed when moving towards the extremities in the sagittal plane (high extension and most importantly, high flexion) and at the most caudally positioned joints (L4L5 and L5S1).

### 3.3 Validation

**FIGURE 6:**
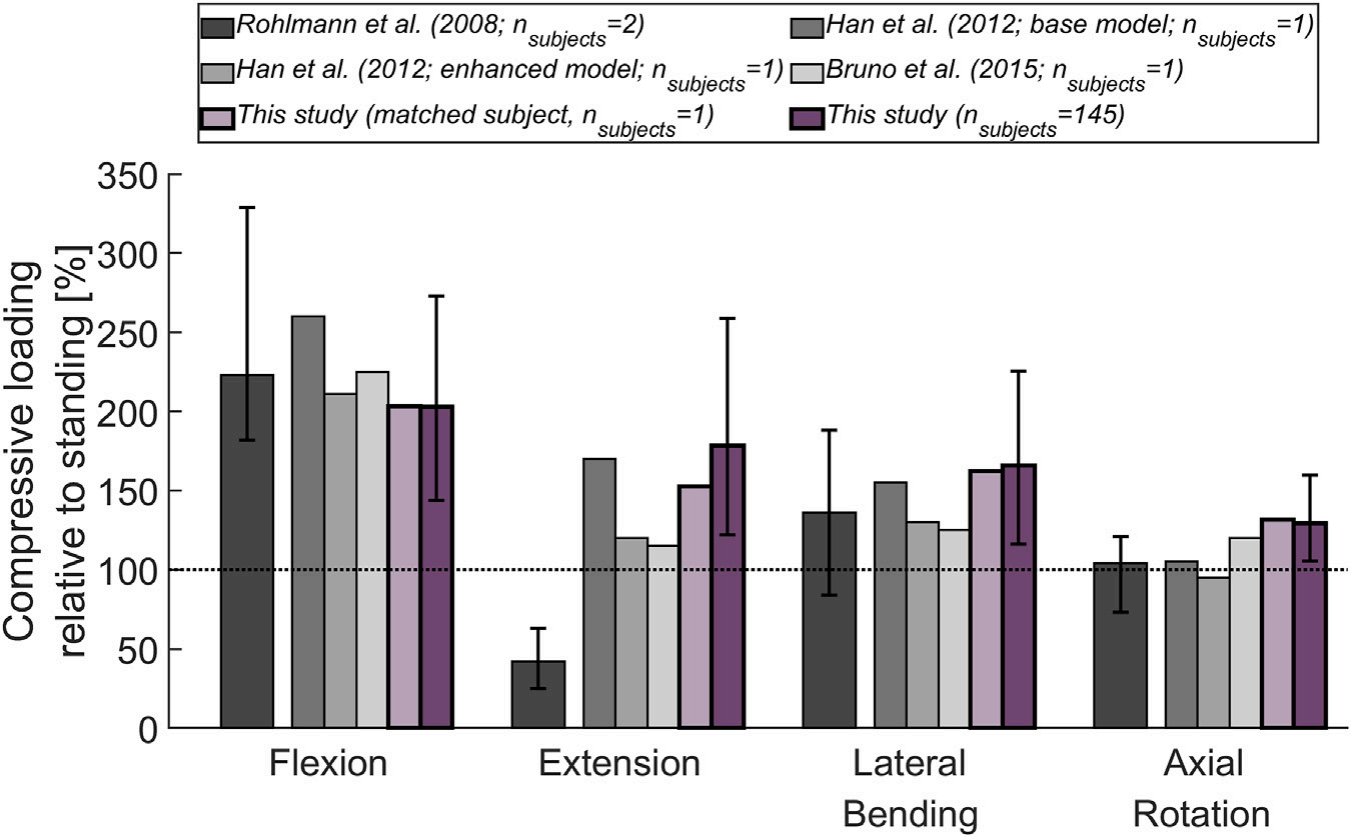
Compressive load at L1L2 (relative to standing position) derived from *in vivo* measurements (Rohlmann et al., 2008) and from previously published thoracolumbar musculoskeletal models (Han et al., 2012; Bruno et al., 2015). These results are being compared to the mean compressive joint load obtained for the 145 subjects considered in this study (dark purple) and for a subject (male, 74 years, 69 kg; light purple) with age and weight properties comparable to those of the subjects in the experiments. The base model of Han et al. (2012) did not incorporate properties derived from passive elements or aspects of muscle dynamics, while these features were added in the enhanced model presented in the same publication. The error bars indicate the range between minimum and maximum values.

**FIGURE 7:**
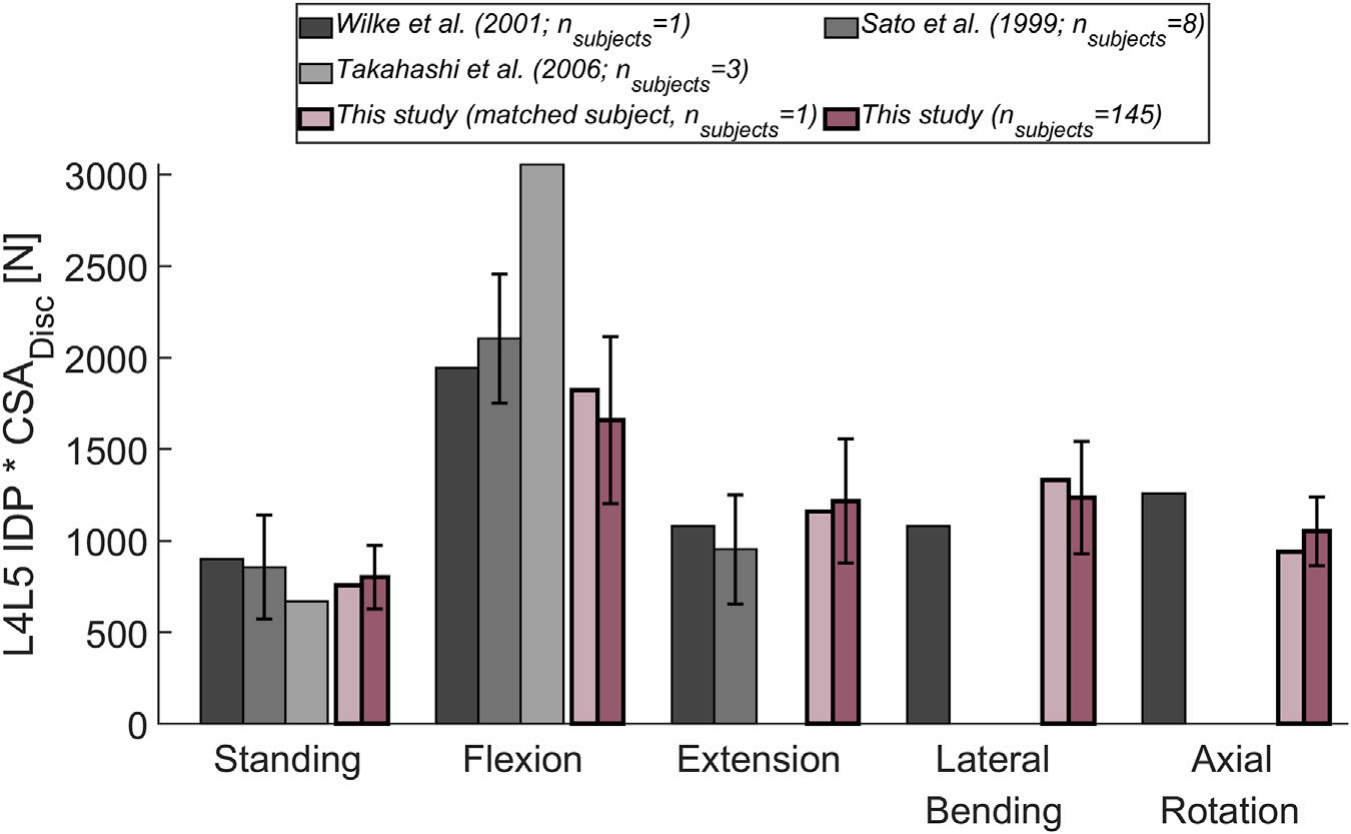
Comparison between measured (Wilke et al., 2001; Sato et al., 1999; Takahashi et al., 2006) and computed IDP at the L4L5 joint. The following upper body positions were simulated: standing, flexion (30°), extension (15°), lateral bending (20°), and axial rotation (30°). To account for a varying CSA of the (mean) L4L5 IVD area in the different experimental studies, the experimentally determined IDP was multiplied by the respective CSA. The obtained results were compared to the mean results of the entire cohort considered in this study (dark purple). Additionally, the results for a subject (male, 34 years, 74 kg; light purple) with weight and age comparable to the subjects in the experimental studies, was depicted. Error bars indicate the standard deviation.

The magnitude of the computed compressive load at the L1L2 joint relative to standing for all subjects was compared to *in vivo* measurements of telemeterized L1 vertebral body implants (Figure 6). The relationship between loading during standing and loading after upper body rotations around the various body axes was similar for the cohort’s average and the single subject matched to the experiment’s participants in terms of sex, age and, weight. Except for flexion, there was a tendency for higher loads to be computed in the simulations, with a considerable discrepancy in extension. The difference between the percentage values obtained with the subject-matched model and the results from the measurements were −20%, 110%, 26%, and 28% for flexion, extension, lateral bending, and axial rotation, respectively. These differences were comparable to those obtained with previously published musculoskeletal models replicating the *in vivo* conditions (Han et al. (2012); Bruno et al. (2015). Figure 7 depicts IDP-related loading of the L4L5 disc for measurements and simulations performed at various upper body positions. Overall, the agreement between experimental results and simulations seems to be highest in standing position, while there is some underestimation seen with the simulation in flexion and lateral bending and slight overestimation of loading in extension and lateral bending. As shown in Figure 7, measured and estimated IDP values were similar, but there was a trend for the computed results to slightly overpredict the pressure within the disc. In terms of compressive loading in the joints of the lower spine, the simulation results obtained in the current study are similar to those of previously published musculoskeletal models (Bassani et al. (2017); Ignasiak et al. (2016a); Bruno et al. (2017), Figure 8). Good agreement was achieved between the results of the study of Bruno et al. (2017) and those computed in this study for the compression at the L3L4 joint.

**FIGURE 8:**
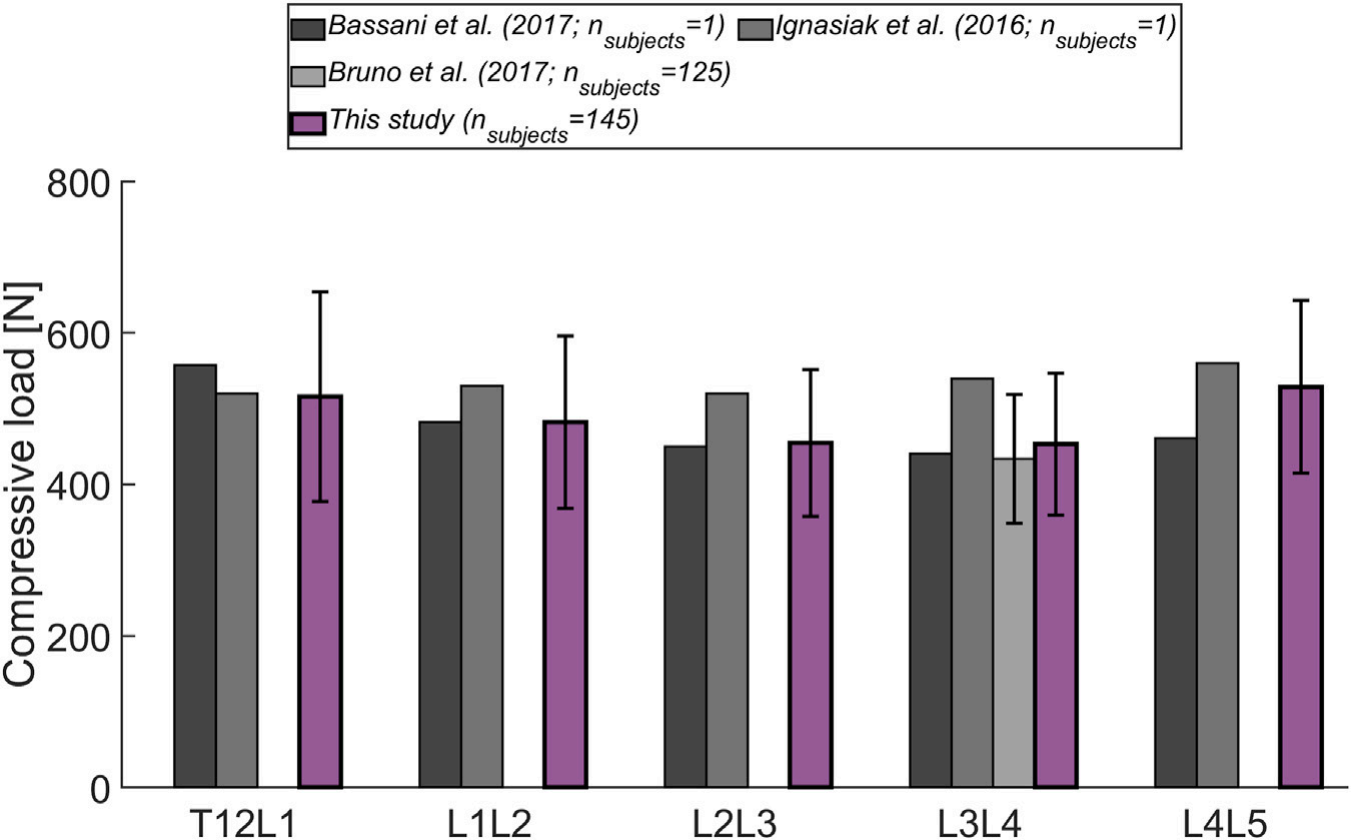
Level-dependent comparison of the magnitude of compressive forces during standing with the outcome from other musculoskeletal models (Bassani et al., 2017; Ignasiak et al., 2016a; Bruno et al., 2017). Error bars indicate the standard deviation.

## 4 Discussion

Previous studies on musculoskeletal models showed that biomechanical loads change considerably with spine alignment and tissue dimensions, along with a person’s height and weight (Han, 2013; Bassani et al., 2017). Moreover, anatomical differences between male and female spinopelvic structures (pelvis, mass distribution, shape of lumbar curvature), as well as age-dependent variations, can be expected to affect the loading magnitude and distribution (Fon et al., 1980; Roussouly et al., 2005; Hay et al., 2015; Bassani et al., 2019). This highlights the necessity to accurately render these aspects when modeling the human spine. We, therefore, developed and validated a tool for the automatized generation of musculoskeletal models incorporating subject-specific alignment and mass distribution based on EOS images.

In contrast to CT and conventional X-ray imaging, the EOS system provides full-body scans in a weight-bearing posture with significantly lower radiation exposure (Dietrich et al., 2013). The use of bi-planar radiographs for musculoskeletal modeling is particularly favorable because these images are frequently acquired in clinical practice for the assessment of spine alignment.

To test the pipeline, models were generated for a cohort of 145 subjects without relevant spine deformations in the frontal plane. The results from the intra-rater and inter-rater reliability assessment of landmark positioning suggest that overall, the model properties and results can be well reproduced with the current annotation procedure (Koo and Li, 2016). The selected subjects formed a diverse cohort in terms of age, weight, and alignment (Table 2; Figure 3). This showed the robustness of the model creation approach and the cohort heterogeneity was reflected in the considerable range of computed joint loads (Figure 4). Furthermore, the average distance of COM from the corresponding vertebral centroid between our computations and the measurements of Pearsall et al. (1996) diverged towards the caudal and cranial ends of the thoracolumbar spine (Figure 3B). Our approach for determining the volume of transversal body sections was based on a simplification, namely fitting ellipses through just four landmarks delimiting the body extremity towards anterior, posterior, left, and right. However, there was also a large discrepancy in sample size, since Pearsall et al. (1996) only took measurements from four subjects, which might have limited the generalizability of their observations. Another factor limiting the comparability of relative COM position is the difference in posture during image acquisition (standing for the EOS images compared to supine in the CT scanner). Finally, this study focused on variations of alignment in the sagittal plane but the presented approach can be expected to similarly capture the fallout from alignment anomalies in the frontal plane (i.e. of scoliotic spines).

For model validation, mean results for the considered cohort were compared to normalized values from *in vivo* and *in situ* studies. The substantial deviation between measured and computed joint load in extended position (Figure 6) has already observed in other *in situ* studies (Han et al., 2012; Bruno et al., 2015). We hypothesize that this is caused by the load-carrying capacity of the facets, which might become more relevant during extension and hence, lead to a reduction of load exerted on the implant. The musculoskeletal models capture the force acting on the whole vertebra and do not differentiate between load transfer through posterior and anterior vertebral structures. Moreover, in addition to corpectomies at the L1 vertebra, the patients in the study of Rohlmann et al. (2008) had posterior spinal fixators in place, possibly causing parts of the load to be transferred across these additional implants. Furthermore, Rohlmann et al. (2008) did not specify the range of motion corresponding to the reported joint load. The level-dependent compressive loads agreed with the values derived from other musculoskeletal models (Figure 8). Also, the relative difference in loading observed between the joints was similar to the trend seen by Bassani et al. (2017) (highest forces were detected at the extremities of the lumbar spine).

Our study had several limitations. The analysis neglected thorax flexibility. As opposed to Bruno et al. (2015), the thorax was modeled as a rigid body and could therefore not account for relative rotations and joint reaction forces acting on the single thoracic vertebrae. However, according to Ignasiak et al. (2016b), this assumption does not considerably affect loading predictions in the lumbar spine. Further, the actual location of the COR hardly corresponds to the center of the intervertebral space, and the restriction of translational degrees of freedom through the use of spherical joints is a simplification. Rather, the COR in the lumbar spine has been described to be positioned more posteriorly and caudally with respect to the (upper) vertebral body. Moreover, the COR is not fixed but drifts during movement relative to the surrounding bony structures (Aiyangar et al., 2017). However, results obtained with another multi-rigid body model and sensitivity analysis performed with our model indicate that slight shifts in COR have no major influence on the results (Senteler et al., 2018). In this study, only the spine alignment in the frontal and sagittal plane was reproduced in the models, possible rotations of bony structures within the transverse plane were not taken into account. The impact on the results of this simplification is the subject of future investigations. Also, muscle properties were not derived from patient-specific measurements. The possibility to improve the models by incorporating image-based information or by using previously published regression models for the prediction of muscle parameters based on subject specifications (for example based on sex, age, height, and weight as proposed by Anderson et al. (2012)) needs to be assessed with a sensitivity analysis. So far, the lumbo-pelvic rhythm was not considered and we did not model the intra-abdominal pressure. According to previous investigations, the latter simplification, may have lead to an overestimation of joint loading (Arshad et al., 2016; Liu et al., 2019). Finally, the impact of passive structures (ligaments, intervertebral discs, facet joints) on spine behavior was not taken into account. It has been shown that the contribution to spine stabilization from these tissues becomes especially relevant at positions further away from the neutral posture (Widmer et al., 2020). Consequently, when optimizing muscle activity at upper body positions increasingly further away from the standing position (0° around all axes), the aforementioned drawback can be expected to have a detrimental effect on the results. We, therefore, refrained from optimizing muscle activity at rotation angles greater than 30° around any axis. Despite these common modeling limitations, the framework represents a substantial advance in patient-specific modeling of the upper body and is likely to reveal novel insights into the biomechanics of the healthy and pathological spine.

## 5 Conclusion

Results obtained with spine multi-rigid body simulations are influenced by 1) the properties of the muscles, 2) the alignment of the CORs, and 3) the arrangement of the segments’ COMs relative to the respective CORs. The present work showed that valuable information on subject-specific aspects concerning features 2) and 3) can be consistently gathered from EOS images. The modeling approach provides a robust tool for the automatized generation of individualized musculoskeletal models, importantly with accurate rendering of alignment and mass distribution. This powerful, high-throughput framework now enables the investigation of a variety of relevant clinical questions concerning the (lower) spine. Our overall aim is to enable studies on the impact of biomechanical aspects on the etiology and progression of pathologies and to perform subject-specific risk assessments. Specifically, we plan to evaluate the link between spine alignment and kinetics together with the possible clinical implications arising from this association.

## Data Availability

The datasets presented in this article are not readily available because they are patient data.
