# Supplementary material for: Subject-Specific Alignment and Mass Distribution in Musculoskeletal Models of the Lumbar Spine
Source: Front Bioeng Biotechnol. 2021 Aug 31;9:721042. doi: 10.3389/fbioe.2021.721042 (PMC8438119; doi:10.3389/fbioe.2021.721042)
Supplement: Supplementary file 1 [file DataSheet1.pdf]

Supplementary Material

	Number of subjects	Age [years]	Weight [Kg]	IVD CSA [mm^2]	Study Type
**Compression Froce:**					
Rohlmann et al. (2008)	2 males	62-71	66-72		*In vivo*
Han et al. (2012)	Based on Rohlmann et al. (2008)	Based on Rohlmann et al. (2008)	Based on Rohlmann et al. (2008)		*In silico* (Anybody)
Bruno et al. (2015)	Based on Rohlmann et al. (2008)	Based on Rohlmann et al. (2008)	Based on Rohlmann et al. (2008)		*In silico* (Opensim)
**IDP:**					
Wilke et al. (2001)	1 male	45	72	1800	*In vivo*
Sato et al. (1999)	8 males	25 (22-29)	73 (60-96)	1590 (1340-1890)	*In vivo*
Takahashi et al. (2006)	3 males	25 (24-26)	72 (70-77)	1910 (1730-2120)	*In vivo*
***In Silico*:**					
Bassani et al. (2017)	1 male	16	47		*In silico* (Anybody)
Ignasiak et al. (2016)	1 male		75		*In silico* (Anybody)
Bruno et al. (2017)	125 males	65 (41-88)	85 (57-123)		*In silico* (Opensim)

Table S1. Published work used for validation of our patient-specific musculoskeletal models. The publications are divided into three groups: those used for comparison of compressive forces acting on L1L2 joints at various upper body positions, those evaluating interdiscal pressures (IDP), and *in silico* studies assessing joint reaction forces in standing position at different joints. Information about number of subjects, age, weight, cross-sectional area (CSA) of the L4L5 intervertebral discs (IVD), and the study type, are listed. Depending on the available information, mean values and/or ranges of values (minimum-maximum) are listed.

	Intra-rater reliability:		Inter-rater reliability:	
	**ICC**	**95% CI**	**ICC**	**95% CI**
**Alignment/Weight Estimation:**				
Pelvic Incidence	0.83	0.60-0.93	0.86	0.64-0.95
Sagittal Vertical Axis	0.99	0.98-1.00	0.98	0.94-0.99
Sacral Slope	0.72	0.41-0.88	0.72	0.42-0.88
Pelvic Tilt	0.98	0.94-0.99	0.97	0.81-0.99
Lumbar Lordosis	0.89	0.74-0.96	0.91	0.77-0.97
Thoracic Kyphosis	0.97	0.93-0.99	0.90	0.77-0.96
Weight Estimation	0.96	0.89-0.98	0.95	0.88-0.98
**Model Output:**				
JRF in Standing Position	0.92	0.50-0.99	0.90	0.38-0.99
Erector Spinae Muscle Tension	0.93	0.49-0.99	0.91	0.40-0.99

Table S2. ICC and 95% CI for intra-rater and inter-rater reliability of radiographs annotation. Alignment, weight estimation, and simulation results were compared.

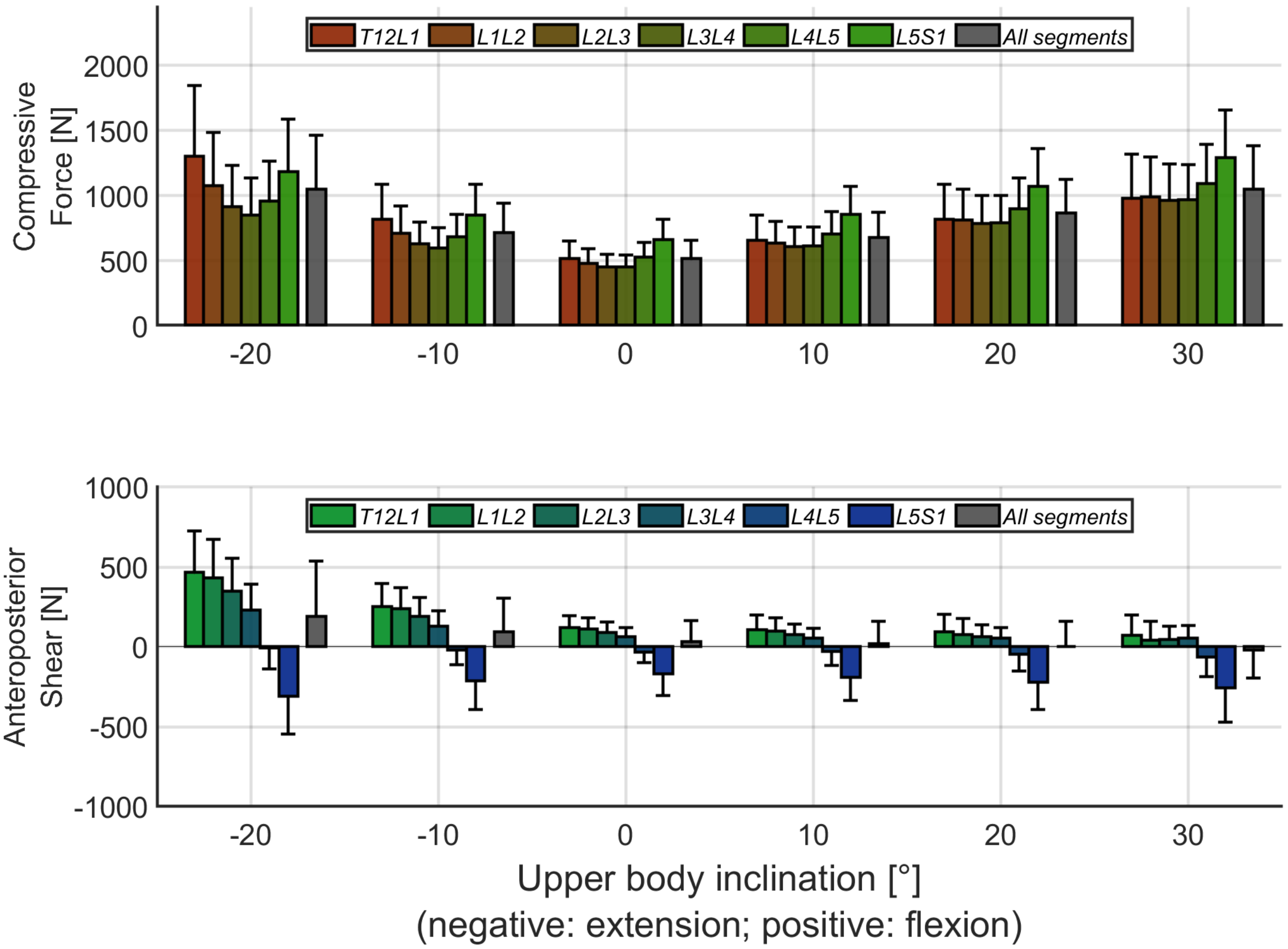

Figure S1. Mean magnitude of the compressive and the anteroposterior shear components of the JRFs during flexion-extension movement of the upper body. The error bars indicate the standard deviation.

REFERENCES

Bassani, T., Ottardi, C., Costa, F., Brayda-Bruno, M., Wilke, H.-J., and Galbusera, F. (2017). Semiautomated 3D Spine Reconstruction from Biplanar Radiographic Images: Prediction of Intervertebral Loading in Scoliotic Subjects. *Frontiers in Bioengineering and Biotechnology* 5. doi:10.3389/fbioe.2017.00001

Bruno, A. G., Bouxsein, M. L., and Anderson, D. E. (2015). Development and Validation of a Musculoskeletal Model of the Fully Articulated Thoracolumbar Spine and Rib Cage. *Journal of Biomechanical Engineering* 137, 081003. doi:10.1115/1.4030408

Bruno, A. G., Mokhtarzadeh, H., Allaire, B. T., Velie, K. R., De Paolis Kaluza, M. C., Anderson, D. E., et al. (2017). Incorporation of ct-based measurements of trunk anatomy into subject-specific musculoskeletal models of the spine influences vertebral loading predictions. *Journal of Orthopaedic Research* 35, 2164–2173

Han, K.-S., Zander, T., Taylor, W. R., and Rohlmann, A. (2012). An enhanced and validated generic thoraco-lumbar spine model for prediction of muscle forces. *Medical Engineering & Physics* 34, 709–716. doi:10.1016/j.medengphy.2011.09.014

Ignasiak, D., Dendorfer, S., and Ferguson, S. J. (2016). Thoracolumbar spine model with articulated ribcage for the prediction of dynamic spinal loading. *Journal of Biomechanics* 49, 959–966. doi:10.1016/j.jbiomech.2015.10.010

Rohlmann, A., Graichen, F., Kayser, R., Bender, A., and Bergmann, G. (2008). Loads on a Telemeterized Vertebral Body Replacement Measured in Two Patients. *Spine* 33, 1170–1179. doi:10.1097/BRS.0b013e3181722d52

Sato, K., Kikuchi, S., and Yonezawa, T. (1999). In Vivo Intradiscal Pressure Measurement in Healthy Individuals and in Patients With Ongoing Back Problems:. *Spine* 24, 2468. doi:10.1097/00007632-199912010-00008

Takahashi, I., Kikuchi, S.-i., Sato, K., and Sato, N. (2006). Mechanical load of the lumbar spine during forward bending motion of the trunk–a biomechanical study. *Spine* 31, 18–23

Wilke, H.-J., Neef, P., Hinz, B., Seidel, H., and Claes, L. (2001). Intradiscal pressure together with anthropometric data – a data set for the validation of models. *Clinical Biomechanics* 16, S111–S126. doi:10.1016/S0268-0033(00)00103-0
